# Key drivers of at‐vessel mortality in demersal sharks

**DOI:** 10.1111/cobi.70100

**Published:** 2025-07-03

**Authors:** David Ruiz‐García, Claudio Barría, Juan A. Raga, David March

**Affiliations:** ^1^ Unidad de Zoología Marina, Instituto Cavanilles de Biodiversidad y Biología Evolutiva Universitat de València Paterna Spain; ^2^ Association for the Study and Conservation of Elasmobranchs and Its Ecosystems (Catsharks) Barcelona Spain; ^3^ Department of Functional Biology, Genetics University of Oviedo Oviedo Spain; ^4^ Centre for Ecology and Conservation, College of Life and Environmental Sciences University of Exeter Penryn UK

**Keywords:** boosted regression trees, bottom trawl, bycatch mitigation, elasmobranch, fisheries management, handling practices, árboles de regresión potenciada, arrastre de fondo, elasmobranquios, gestión de pesquerías, mitigación de la pesca incidental, prácticas de manejo

## Abstract

Chondrichthyans are highly vulnerable to fisheries overexploitation, and postcapture mortality poses a significant threat to most species. Global bycatch mitigation guidelines recommend adopting hierarchical decision‐making approaches tailored to species‐specific vulnerabilities and socioeconomic and regulatory contexts. Effective implementation of such strategies requires robust understanding of the factors driving vulnerability to postcapture mortality. To address this need, we developed a machine learning method to identify key drivers of at‐vessel mortality (AVM) based on a broad set of biological, environmental, and fishing‐related parameters. We sought to reveal interactions among predictors, nonlinear responses between these variables and mortality risk, and threshold values beyond which the likelihood of mortality increased markedly. We applied this approach to trawl bycatch data on small‐spotted catshark (*Scyliorhinus canicula*) and blackmouth catshark (*Galeus melastomus*) in the western Mediterranean. Body size, air temperature, and on‐deck time emerged as the primary AVM drivers. Mortality risk increased substantially at temperatures above 20°C for *S. canicula* and 16°C for *G. melastomus*, with on‐deck exposure exceeding 15 min, and when body size was below 40 and 55 cm, respectively. Identification of these drivers and thresholds provides valuable insights for bycatch mitigation; can inform strategies for more threatened, closely related, or physiologically and ecologically similar species; and may support management authorities in adopting targeted bycatch avoidance strategies, gear selectivity, and mortality reduction measures. Such measures can be tailored to specimens, areas, and periods of heightened mortality risk to maximize effectiveness. Furthermore, our scalable modeling approach offers a robust tool for identifying critical AVM drivers across regions and species, and its applicability can be extended to broader fisheries management and global conservation efforts.

## Graphical abstract

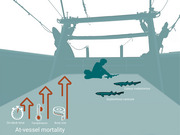

## Abstract (es)

Factores principales de la muerte de tiburones demersales a bordo de barcos

**Resumen:**

Los condrictios son muy vulnerables a la sobreexplotación pesquera, y la mortalidad posterior a la captura supone una amenaza importante para la mayoría de las especies. Las directrices mundiales sobre la mitigación de la pesca incidental recomiendan adoptar enfoques jerárquicos de toma de decisiones adaptados a las vulnerabilidades específicas de cada especie y a los contextos socioeconómicos y normativos. La aplicación eficaz de tales estrategias requiere una comprensión sólida de los factores que determinan la vulnerabilidad a la mortalidad post‐captura. Para responder a esta necesidad, desarrollamos un método de aprendizaje automático para identificar los factores clave de la mortalidad post‐captura con base en un amplio conjunto de parámetros biológicos, ambientales y relacionados con la pesca. Intentamos revelar interacciones entre predictores, respuestas no lineales entre estas variables y el riesgo de mortalidad, y valores del umbral a partir de los cuales la probabilidad de mortalidad aumentaba notablemente. Aplicamos este planteamiento a los datos de capturas accesorias en redes de arrastre de *Scyliorhinus canicula* y *Galeus melastomus* en el Mediterráneo occidental. La talla, la temperatura del aire y el tiempo de permanencia en cubierta resultaron ser los principales factores determinantes de la mortalidad a bordo. El riesgo de mortalidad aumentó sustancialmente a temperaturas superiores a 20 °C para *S. canicula* y 16 °C para *G. melastomus*, con una exposición en cubierta superior a 15 minutos, y cuando la talla era inferior a 40 cm y 55 cm, respectivamente. La identificación de estos factores y umbrales proporciona información valiosa para la mitigación de la pesca incidental; puede servir de base a estrategias para especies más amenazadas, estrechamente relacionadas o fisiológica y ecológicamente similares; y puede ayudar a las autoridades gestoras a adoptar estrategias específicas para evitar la pesca incidental, selección de equipo y medidas de reducción de la mortalidad. Estas medidas pueden adaptarse a los especímenes, las zonas y los periodos de mayor riesgo de mortalidad para maximizar su eficacia. Además, nuestro enfoque de modelo escalable ofrece una herramienta robusta para identificar los factores críticos de mortalidad a bordo en todas las regiones y especies, y su aplicabilidad puede extenderse a la gestión de pesquerías más amplias y a los esfuerzos globales de conservación.

## INTRODUCTION

Chondrichthyans (sharks, rays, and chimaeras) play a key role in the regulation of marine ecosystems by controlling prey population dynamics and maintaining trophic connectivity across habitats (Dedman et al., 2024). However, their slow life‐history traits (low fecundity, late maturation, and extended lifespans) make them highly vulnerable to anthropogenic pressures, primarily driven by fishing exploitation (Dulvy et al., 2021). At a global scale, over one third of the species are considered threatened according to the International Union for Conservation of Nature (IUCN) Red List criteria (Dulvy et al., 2024, 2021). Although capture of 74% of species is considered unintentional, many are regularly retained for commercial purposes. Some species have become unofficial targets. That is, species that are not formally recognized as targets in management plans have gained substantial commercial value and represent a significant source of income (Dulvy et al., 2021). Yet, most countries lack science‐based catch limits or bycatch reduction mandates targeting chondrichthyans (Koehler et al., 2025; Pacoureau et al., 2023).

Due to widespread population declines and the lack of robust stock assessments, authorities with chondrichthyans conservation mandates, particularly the European Union (EU) and Regional Fishery Management Organizations (RFMOs), have primarily relied on retention bans to address overexploitation; fishing quotas are employed less frequently (Gilman et al., 2024; Giovos et al., 2024). Although these are often vital to disincentivize targeted fishing and simplify enforcement, they do not address the mortality associated to accidental captures, which remains a major conservation concern (Finucci et al., 2024; Koehler et al., 2022). Fisheries postcapture mortality extends beyond the retained specimens to include those discarded dead (i.e., at‐vessel mortality [AVM]) and those discarded alive that do not survive (postrelease mortality). Discard mortality is especially relevant for nontarget or low‐valued species, including protected species, which are frequently or routinely discarded (Ellis et al., 2024, 2017). Unabated discard mortality remains a major risk to population sustainability, underscoring the need for comprehensive bycatch mitigation strategies.

Global nonbinding initiatives to guide chondrichthyan bycatch mitigation, such as the International Plan of Action for Conservation and Management of Sharks (IPOA‐sharks), recommend adopting hierarchical decision‐making frameworks to tailor the use of measures to specific contexts, generally categorized into 3 major groups: capture prevention, escape facilitation, and postcapture mortality reduction (Drynan & Baker, 2024; Ellis et al., 2024; Gilman et al., 2024). Prioritizing strategies based on species‐specific catchability, postcapture mortality rates, and socioeconomic constraints has proven to be the most effective approach, balancing feasibility with conservation impact (Ellis et al., 2024; Gupta et al., 2020; Pacoureau et al., 2023). Consequently, accurate AVM estimates are essential not only for developing quantitative stock assessments and population viability models but also for developing targeted mitigation measures (Gilman, Hall, et al., 2022). However, the effectiveness of these measures also depends on how they are integrated into regulatory frameworks (Koehler & Lowther, 2025; Pacoureau et al., 2023).

The EU has recently launched an action plan for “protecting and restoring marine ecosystems for sustainable and resilient fisheries” aiming to achieve its goals by 2030 under its Common Fisheries Policy and Marine Strategy Framework Directive. The plan calls on member states, along with the European Parliament and Council, to implement measures aimed at improving gear selectivity and reduce fisheries impact on sensitive species (European Commission, 2023). Bycatch management within the EU operates under a complex regulatory framework comprising international binding instruments, EU directives, RMFOs, and national policies (Giovos et al., 2024; Koehler et al., 2025). Bycatch mitigation efforts rely on spatiotemporal closures and protected areas, often targeting ecologically important regions but frequently leaving key areas beyond coastal waters unprotected (European Union, 2023; Hilborn et al., 2022; Jacquemont et al., 2024). Escape mechanisms based on bycatch reduction devices have demonstrated potential but remain mandatory in only a few fisheries (European Union, 2022; Huang et al., 2024). Mortality reduction strategies, including best handling and release practice guidelines, have been promoted by international institutions, including the FAO, RFMOs, and EU member states. However, these guidelines are generally nonbinding (mandatory measures limited to the quick release of protected species), and challenges to implementation and compliance remain (European Union, 2022; Wosnick et al., 2023).

Despite this set of measures, gaps to reduce bycatch mortality persist due to their largely nonmandatory nature, inconsistences in implementation or compliance, and insufficient data on the factors influencing AVM risk. These factors hinder the development of tailored and effective mitigation strategies (Ellis et al., 2024; Gilman et al., 2024; Giovos et al., 2024). In this context, understanding the drivers of AVM for chondrichthyans is crucial to align mitigation strategies with species vulnerabilities and fishery‐specific constraints and will offer insights applicable to regions facing similar conservation and management challenges (Gilman, Hall, et al., 2022). However, AVM data remain scarce for most species, with rates varying depending primarily on their ability to withstand fisheries‐induced trauma and stress (Ellis et al., 2017; Gilman, Chaloupka, et al., 2022). Phylogenetically linked biological and ecological traits—including body size, metabolic capacity, gill ventilation mode, and behavioral responses to capture—have been associated with differences in mortality risk among species (Dapp et al., 2016; Ellis et al., 2017; Gilman, Chaloupka, et al., 2022). At intraspecific levels, AVM rates are further influenced by individual biological traits, fishing operation characteristics, and environmental conditions at the time of capture (Barragán‐Méndez et al., 2019; Braccini & Waltrick, 2019; Prado et al., 2022).

Despite growing recognition of AVM drivers, studies have primarily examined each of these factors individually (e.g., Guida et al., 2016; Knotek et al., 2022; Prohaska et al., 2021; Talwar et al., 2017). Holistic approaches that assess their combined effects remain scarce in the scientific literature. To fill this knowledge gap, modeling frameworks are essential for evaluating the relative influence of multiple AVM drivers and accounting for nonlinear effects and interactions (Gilman, Chaloupka, et al., 2022). These frameworks can aid management authorities in identifying specimen‐ and species‐specific vulnerabilities and refining regulatory decisions tailored to distinct fishing contexts, ultimately contributing to enhance bycatch mitigation plans.

We analyzed fisheries‐dependent trawl data from the western Mediterranean to assess the biological, environmental, and fishing‐related drivers influencing shark AVM rates. To address data limitations in AVM research, studying model taxa can provide transferable insights to guide broader conservation strategies. We focused on the small‐spotted catshark (*Scyliorhinus canicula* [Linnaeus, 1758]) and the blackmouth catshark (*Galeus melastomus* [Rafinesque, 1810]), 2 of the most frequently captured chondrichthyan species across EU waters (Ruiz‐García et al., 2023). Both are classified as least concern based on IUCN Red List criteria. Although not currently threatened, these species require continued monitoring due to their low yet existing commercial value, shifting seafood markets, and the depletion of other target stocks, compounded by the lack of targeted management measures in EU waters for these species (Colloca et al., 2024; Giovos et al., 2024; Koehler et al., 2025). Their shared traits—small body size, prolific life histories, and buccal pumping ventilation—and contrasting depth preferences make them a valuable comparative model for broader applicability to other threatened species (Pardo & Dulvy, 2022).

In assessing the AVM dynamics in these model species, we aimed to provide critical insights for informing conservation strategies for more threatened chondrichthyans and to address key knowledge gaps on mortality drivers. Using a machine learning modeling approach, we evaluated the combined influence of a comprehensive set of biological, environmental, and fishing‐related drivers on the probability of AVM, explicitly accounting for interactions and nonlinear effects to identify mortality thresholds. We aimed to develop a scalable framework to identify primary AVM drivers applicable across species and regions that would provide insights and tools for management authorities (e.g., the EU, RFMOs, or national agencies). We also sought to identify bycatch mitigation measures that would effectively reduce discard mortality, particularly within the scope of current EU Action Plan, and that could be applied to broader fisheries management efforts.

## METHODS

### Study fishery and data collection

**FIGURE 1:**
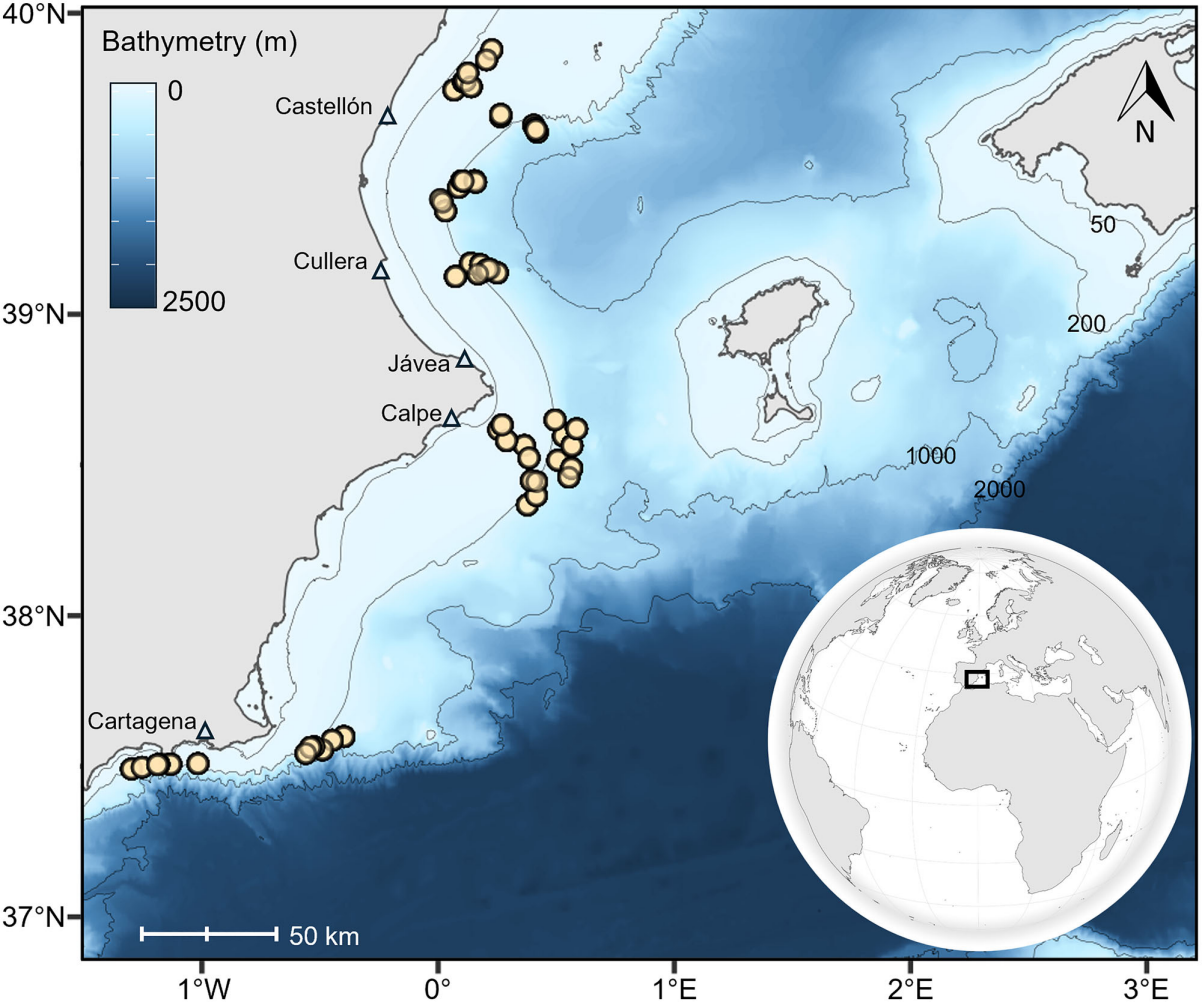
Position of the 66 fishing tows (yellow circles) analyzed in a study of at‐vessel mortality in demersal chondrichthyans (blue triangles, 5 home ports of the tows from which observation campaigns were conducted).

AVM data were collected during observation campaigns on board commercial bottom trawlers in the western Mediterranean. The activity was conducted under the auspices of the Regulation of the European Parliament and of the Council for Fishing in the General Fisheries Commission for the Mediterranean (GFCM) Agreement area and amending Council Regulation (EC) 1967/2006. Our work did not require ethical review and approval. This fishery operates in northern Spain, established by the GFCM, and overlaps with 3 important shark and ray areas (ISRAs) designated by the IUCN in part because of its significance for our study species (Jabado et al., 2023). A total of 66 tows were examined on board 8 different commercial fishing vessels from 5 different home ports, which operate across the continental margin (Figure 1). Observation campaigns were conducted seasonally from December 2020 to June 2022 at each of the studied ports. A stratified random sampling was conducted to ensure a proportional distribution of tows across the fisheries in operation: hake (*Merluccius merluccius*), mullet (*Mullus* spp.), and deep‐water rose shrimp (*Parapenaeus longirostris*) on the continental shelf (50–250 m); Norway lobster (*Nephrops norvegicus*) on the upper slope (200–600 m); and the blue and red shrimp (*Aristeus antennatus*) on the lower slope (400–800 m) (Ruiz‐García et al., 2023).

The geographic position of the vessels was tracked using a GPS (Garmin Ltd, EEUU). The SCANMAR system (Catch Control Systems, Scanmar AS) was used to monitor fishing operations by determining the moments when the trawl reached and left the seabed, measuring the effective towing duration and average towing depth. The towing depth ranged from 50 to 737 m, and the effective tow duration ranged from 0.8 to 7.0 h (mean [SD] = 3.2 h [1.5]). Fishing gear consisted of a bottom otter trawl design fitted with a squared 40‐mm cod‐end mesh. Trawling occurred mostly over soft bottoms, including sandy and muddy substrates. The total biomass captured in the cod‐end was measured after the catch was sorted in fishing bins. The environmental conditions to which specimens were exposed during their time on deck were also monitored. The state of the sea was measured using the Douglas scale, wind strength with the Beaufort scale, and cloud cover with the Oktas scale, which was transformed into percentages.

All the specimens of *S. canicula* and *G. melastomus* or a representative random sample for each tow was counted, sexed, and measured to the nearest millimeter. Maturity was estimated using length at first maturity (L_50_) indices available for the study region (40.04 and 38.74 cm for *S. canicula* females and males, respectively, and 51.92 and 49.91 cm for *G. melastomus* females and males, respectively [Ramírez‐Amaro et al., 2020]).

AVM was assessed at the time when sharks were released back to sea without delaying or speeding up the conventional timing of the commercial discarding practices. When the specimens were instead commercialized, the assessment occurred when they were sorted and selected. The time on deck was measured as the time since the cod‐end was lifted from the sea surface until the specimen was released or processed. A specimen was considered dead when it exhibited *rigor mortis* or was completely limp with no activity or response to stimuli, including jaws hanging open even when tactilely stimulated (Braccini et al., 2012). When a specimen did not exhibit either of these patterns, it was classified as alive.

### Predictor variables

A set of predictors was selected to assess the primary drivers of AVM for *S. canicula* and *G. melastomus* (Table 1). These predictors encompassed biological traits, fishing practices, and environmental conditions. The selection of predictors was based on prior studies in which health status and postcapture mortality of chondrichthyans were analyzed (e.g., Barragán‐Méndez et al., 2019; Braccini & Waltrick, 2019; Prado et al., 2022; Revill et al., 2005). Biological predictors were body size (TL), maturity (MAT), and sex (SEX). Environmental drivers were state of the sea (SEASTATE), wind strength, cloud cover, atmospheric temperature (ATEMP), and the difference between atmospheric temperature and sea bottom temperature (DTEMP). Predictors related to fishing operations were tow depth (DEPTH), towing speed (SPEED), towing duration (DUR), total catch biomass in the cod‐end, and time exposed on deck (DECKTIME). Fishery type was not included as a predictor because operational differences in fisheries were considered within the fishing operation parameters. Additionally, all fisheries primarily targeted shrimp species with similar body structures, unlikely to differentially harm sharks, and species with pronounced spines or teeth were consistently distributed across fishery types. Details on the description of each predictor and environmental data sources are summarized in Table 1.

**TABLE 1:** Predictor variables considered in a model of at‐vessel mortality for *Scyliorhinus canicula* and *Galeus melastomus*.

Predictor	Predictor abbreviation	Description	Temporal resolution	Spatial resolution	Units
Body size	TL	Specimen length from the tip of the snout to posterior end of caudal fin	–	–	cm
Maturity	MAT	Immature or mature based on the length at first maturity (L_50_)	–	–	–
Sex	SEX	Male or female based on the presence or absence of claspers through visual examination	–	–	–
Tow depth	DEPTH	From SCANMAR system included in fishing gear	–	–	m
Effective towing duration	DUR	Time elapsed since gear reached seabed until it started to rise based on the SCANMAR system incorporated in the gear	–	–	min
Towing speed	SPEED	Distance covered during the tow divided by its effective duration and transformed to knots	–	–	kn
Total catch biomass in the tow cod‐end	TOWMASS	Total biomass of all the catch present in the cod‐end	–	–	kg
Time exposed on deck	DECKTIME	Total time elapsed since cod‐end was raised from the sea surface until the specimen was released back to sea or separated for sale	–	–	min
Cloud coverage	CLOUD	Estimated during specimen exposure on deck with the Oktas scale by determining how many eighths of the sky are covered in cloud and transformed to percentage	–	–	%
Sea state	SEASTATE	Estimated during specimen exposure on deck with Douglas scale, which measures the height of the waves and amount of swell in a scale from 0 to 9	–	–	–
Wind force	WIND	Estimated during specimen exposure on deck with the Beaufort scale, which measures the conditions at sea in relation to wind speed in a scale from 0 to 12	–	–	–
Atmospheric temperature	ATEMP	Extracted from ERA5 as reanalysis data from https://climate.copernicus.eu/	Hourly	1/4	°C
Change from atmospheric to sea bottom temperature	DTEMP	Sea bottom temperature (BOTTEMP) extracted from Mediterranean Sea Physics Reanalysis in https://marine.copernicus.eu/; change was calculated as the difference between atmospheric temperature and sea bottom temperature (ATEMP − BOTTEMP)	Hourly	1/24	°C

*Note*: Dash indicates that spatial and temporal scales or units are not applicable to the predictor.

### Analyses of the drivers of AVM

We used boosted regression trees (BRTs) models to assess the effect of biological, environmental, and fishing operation parameters on AVM for *S. canicula* and *G. melastomus*. A BRT model is a machine learning method commonly used to predict habitat preferences (Finucci et al., 2021; March et al., 2021), but it is also used to predict fishing mortality and other population parameters used in stock assessments (Kornis et al., 2019; McClanahan et al., 2022). It constitutes a useful approach for these analyses because it can consider potential interactions between variables and nonlinear relationships (Elith et al., 2008).

Although collinearity among environmental variables does not affect BRT predictions, it can influence the interpretation of the model (Dormann et al., 2013). Thus, collinearity among variables was examined by calculating the Spearman pairwise correlation coefficient, which is robust to nonlinear relationships. The ATEMP and DTEMP showed high correlated values for both species (Spearman correlations = 0.96 and 1 for *S. canicula* and *G. melastomus*, respectively) (Appendix S1). This is expected because DTEMP is relatively stable through time in each sampled location. We kept ATEMP because it was easier to measure and predict and thus provided a readily available tool for estimating mortality. However, considering that the change in temperature may trigger physiological responses leading to mortality, we used a linear regression between ATEMP and DTEMP to provide further insight into the particular change in temperature (DTEMP) that influences AVM. Sea state and wind strength also showed important correlations for *G. melastomus* (Spearman correlations >0.7 [Appendix S1]). In this case, wind strength was kept because it relates to the state of the sea but also may contribute to catch desiccation.

The dismo package in R (R Core Team, 2024) was used to fit one BRT model per species. We used a Bernoulli family, suitable for the binomial response variable with levels dead (1) and alive (0). A BRT model requires the optimization of 4 parameters (Elith et al., 2008): number of trees (boosting iterations), tree complexity, learning rate (shrinkage), and the bag fraction (proportion of data randomly selected at each iteration). Following March et al. (2021), we created the following combinations of potential values for these parameters: number of trees ranging from 50 to 10,000 in 50‐tree increments; tree complexity set to 1, 3, or 5; learning rates of 0.005, 0.001, or 0.01; and bag fractions of 0.5, 0.6, or 0.7. To account for the repeated‐measure structure derived from obtaining multiple samples from each tow, we introduced a block factor in the cross‐validation process (March et al., 2021; Roberts et al., 2017). We used random groups of tows in a 5‐fold cross‐validation, meaning that all data from such tows were excluded from the training data set and used to validate the model. In line with previous recommendations provided by Elith et al. (2008), we chose the combination of parameters that exceeded 1000 trees and minimized the deviance and increased the area under the receiver operating characteristic curve (cross‐validated AUC, a metric for model predictive performance) during cross‐validation. When faced with ties, we prioritized models with higher learning rates, lower tree complexities, and fewer trees to reduce overfitting.

After running the parameter optimization for both species, we selected the parameters highlighted in Appendices S2 and S3 to fit the final models. In BRT, variable selection occurs because the model predominantly disregards noninformative predictors when fitting trees (Elith et al., 2008). However, to explicitly exclude unimportant variables, we included an additional variable with random values between 1 and 100 to serve as an indicator of variables with influence exceeding or falling below that of random (March et al., 2021; Scales et al., 2017). All predictor variables that exhibited influence greater than the random variable were included in the final models. To account for model stochasticity and estimate associated uncertainty in predictions, we adopted a bootstrap approach (Hazen et al., 2018; Hijmans et al., 2023). The model was fitted 100 times, each time by resampling half of the data (with replacement) (Hindell et al., 2020). As a measure of uncertainty, we calculated the 95% confidence interval range.

All analyses were performed with R 4.4.1 (R Core Team, 2024). The R code and data used on these analyses are available in Zenodo public repositories https://doi.org/10.5281/zenodo.11259544 and https://doi.org/10.5281/zenodo.15190183, respectively.

## RESULTS

### AVM rates and predictor data

A total of 1430 specimens of *S. canicula* and 1015 specimens of *G. melastomus* were assessed during the 66 surveyed tows. The overall rate of AVM for *S. canicula* was 27.4%, whereas for *G. melastomus*, it was 80.6%. Discard rates were 91.3% and 87.7% for *S. canicula* and *G. melastomus*, respectively. Retention for commercial sale occurred occasionally for both species, particularly for specimens larger than 40 cm. Shark meat was processed before auction (peeled, eviscerated, and, in some cases, beheaded). The overall mean time to discard was 41.2 min (SD 17.4) (range 5–105 min).

**FIGURE 2:**
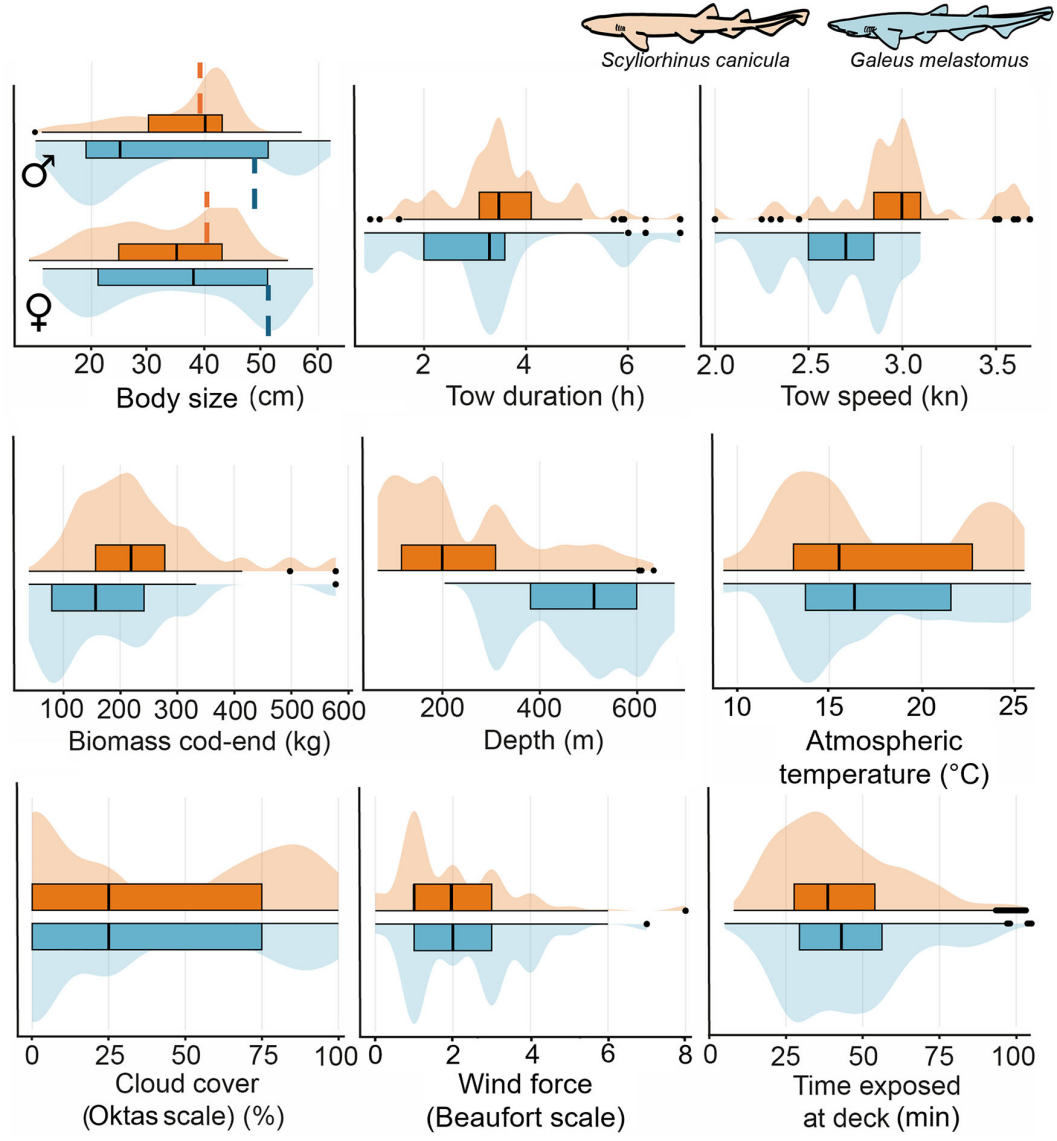
Distribution of key predictor variables included in the analysis of at‐vessel mortality for *Scyliorhinus canicula* (orange) and *Galeus melastomus* (blue) (violin plots: shaded areas, density distribution of each predictor; overlaid boxplots: central vertical lines, medians; box ends, interquartile ranges; whiskers, minimum and maximum values; dashed lines, L_50_ threshold separating immature and mature individuals).

Observed values of predictor variables are in Figure 2. Overall, the studied sample encompassed specimens from newborns to fully grown male and female adults. For *S. canicula*, the sample comprised 592 males and 838 females and overall 820 immature and 610 adult specimens. For *G. melastomus*, it comprised 593 males and 422 females and overall 730 immature and 285 mature specimens.

### Drivers of AVM for *S. canicula*

**FIGURE 3:**
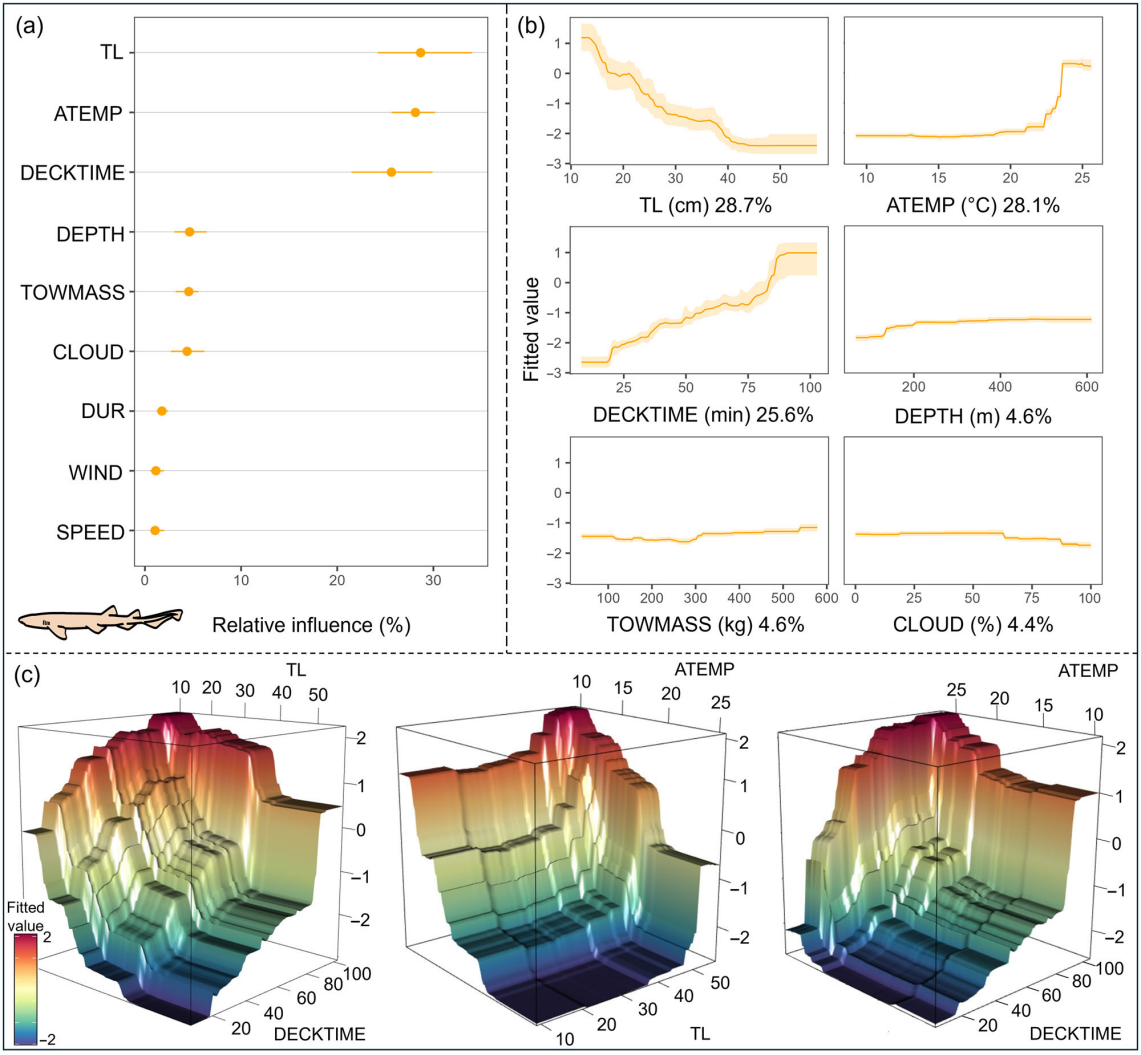
Effect of the biological, environmental, and fishing operation drivers on at‐vessel mortality likelihood for *Scyliorhinus canicula*: (a) predictor relative influence (dots, medians; lines, 95% confidence interval ranges of bootstrap predictions [*n* = 100]); (b) mean relative influence of the 6 best predictors (shading, 95% confidence interval estimated from bootstrap predictions [*n* = 100]); and (c) strongest interactions in the boosted regression trees model (*y*‐axis, fitted value). Abbreviations defined in Table 1.

The evaluation of the model for *S. canicula* through cross‐validation procedures indicated a good fit to the observed data; the model explained 82.1% of the cross‐validated deviance and had a high predictive performance (cross‐validated AUC score = 0.87) (Appendix S2). Body size, followed by ATEMP, and DECKTIME were among the top predictors (relative influence >10%) (all had nonlinear response curves) (Figure 3a,b).

The probability of AVM was higher for smaller specimens and decreased progressively as body size increased. Probability stabilized at approximately 40 cm. The AVM likelihood began to increase progressively after approximately 15 min of exposure on deck. A threshold was reached after 75 min, when the odds of AVM suddenly increased, although some specimens survived up to 1 h and 30 min. AVM likelihood was higher at shorter times on deck for smaller specimens than for the larger ones, which tended to resist for longer periods (Figure 3c). In terms of atmospheric temperature, a threshold was found at approximately 20°C, beyond which AVM probability increased rapidly and stabilized at around 24°C (Figure 3b). Given the stability of temperature in the sea bottom, these atmospheric temperatures corresponded to change from atmospheric to sea bottom temperature of approximately 6°C and 10.5°C, respectively (Appendix S4). AVM likelihood was lower for larger specimens than for smaller ones when exposed to higher atmospheric temperatures (Figure 3c). Higher atmospheric temperatures increased the odds of AVM in specimens that were exposed on deck for larger periods (Figure 3c).

### Drivers of AVM for *G. melastomus*

**FIGURE 4:**
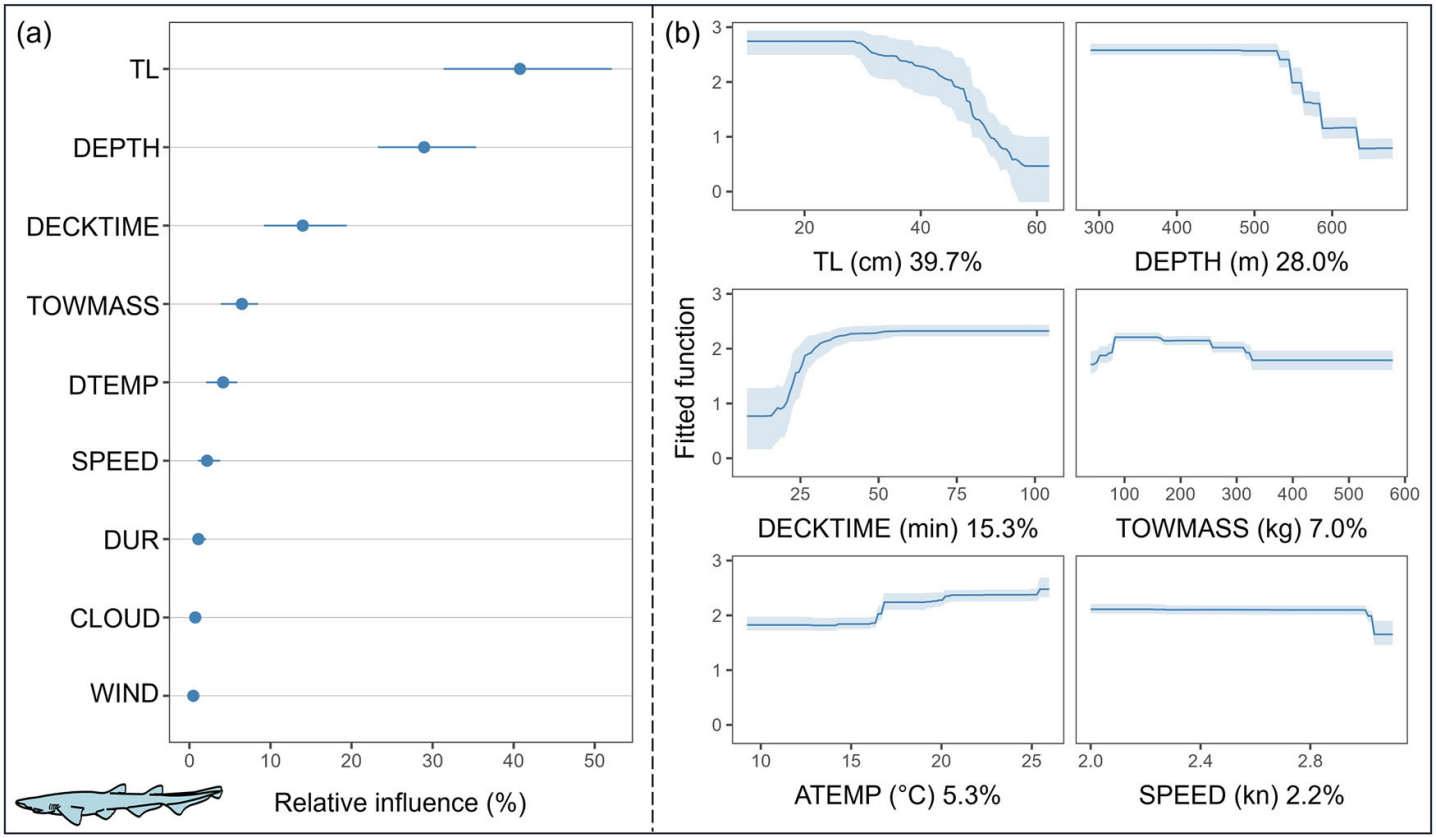
Effect of the biological, environmental, and fishing operation drivers on at‐vessel mortality likelihood for *Galeus melastomus*: (a) predictor relative influence (dots, median; lines, 95% confidence interval range of bootstrap predictions [*n* = 100]) and (b) mean relative influence of the 6 best predictors (shading, 95% confidence interval estimated from 100 bootstrap samples of the data set). Abbreviations defined in Table 1.

The evaluation of the model for *G. melastomus* through cross‐validation procedures indicated a good fit to the observed data. The model explained 76.9% of the cross‐validated deviance and had a high predictive performance (cross‐validated AUC score = 0.87) (Appendix S3). The body size, followed by the fishing depth, and DECKTIME were among the top predictors (relative influence >10%). They exhibited nonlinear response curves (Figure 4a). AVM likelihood was especially pronounced for small specimens and commenced to decrease progressively when individuals were longer than 30 cm. This likelihood stabilized at about 55 cm. It also increased significantly at fishing depths larger than 550 m, approximately. Fishing depth was positively correlated with body size, indicating that larger specimens occurred in the deeper areas of the study area (Appendix S1). A sudden increase in AVM likelihood occurred as time on deck increased, beginning approximately at 15 min after capture and stabilizing approximately at 35 min on deck.

The total biomass in the cod‐end and ATEMP were weaker predictors but accounted for a low but relevant influence on AVM probability (relative influence >5%). The likelihood of AVM varied as biomass in the cod‐end increased (slight increase from 100 to 300 kg). AVM probabilities also increased rapidly above approximately 16°C ATEMP (Figure 4b). Given the stability of temperature in the sea bottom, this atmospheric temperature corresponded to a temperature change of approximately 2.5°C (Appendix S4). No relevant interactions between predictors were found for *G. melastomus*.

## DISCUSSION

Our observation campaigns provide valuable insights into AVM rates and fishing operation conditions in a commercial trawl fishery from the western Mediterranean. Our machine learning framework effectively integrated a comprehensive set of biological, environmental, and fishing operation parameters and achieved high predictive performance for AVM estimation in *S. canicula* and *G. melastomus*. To our knowledge, ours is the first study to apply this methodology while incorporating such a broad range of drivers and thus addresses important knowledge gaps. The results revealed key AVM drivers and identified interactions among predictors and critical thresholds beyond which mortality risk increased greatly. These findings provide valuable insights into species‐ and specimen‐specific factors that affect AVM vulnerability under diverse environmental and fishing conditions.

### AVM rates

Ours is the first study to accurately quantify AVM rates for the deepwater species *G. melastomus*. We found a high mortality rate of 80.6%, which is consistent with previous observations of poor health status in captured specimens (Scacco et al., 2023). The shallow‐dwelling species *S. canicula* exhibited a relatively low AVM rate of 27.4%, consistent with findings from commercial trawl fisheries (Rodríguez‐Cabello et al., 2005). We found important differences in AVM rates between *S. canicula* and *G. melastomus*, despite their similar body size, life histories, ecological traits, and ventilation mode—factors known to influence AVM rates (Dapp et al., 2016). The primary difference between these species is their inhabiting depth ranges, suggesting higher vulnerability for deepwater species. However, the primary drivers influencing AVM at intraspecific level were consistent for both species despite slight differences in the shapes of their dependence curves.

Generally, deepwater chondrichthyans have relatively low metabolic rates as a consequence of adaptations to low temperatures, scarce food supply, increased pressure, low oxygen saturation, and absence of light (Condon et al., 2012; Rigby & Simpfendorfer, 2015). Chondrichthyans possessing a lower metabolic scope exhibit reduced resilience to withstand and recover from elevated stress levels without compromising other aerobic demands (Skomal & Mandelman, 2012). Thus, the return to physiological homeostasis is thought to be delayed and less likely to happen for species dwelling at deeper depths (Prohaska et al., 2021; Skomal & Mandelman, 2012). This was particularly evident in our study. The deepwater species *G. melastomus* faced higher mortality risks than the shallower‐dwelling *S. canicula*. Although evidence remains limited, the rapid and significant change in depth—coupled with associated pressure and temperature shifts—may lead to embolisms caused by the supersaturation of metabolic gases (O_2_, CO_2_, or both) in tissues and to liver damage resulting from alterations in liver oil density and composition (García‐Parraga et al., 2015; Priede et al., 2020). Further research is needed to determine whether these effects are significant contributors to AVM.

### Biological traits

The probability of AVM progressively decreased as body size increased for both species, stabilizing at approximately 40 cm for *S. canicula* and 55 cm for *G. melastomus*. Chondrichthyan physiological responses to capture stress are closely tied to their body size, especially those related to glucose dynamics (Braccini & Waltrick, 2019; Skomal & Mandelman, 2012). Large specimens generally possess large glycogen stores, which provide glucose during the fight response, which allows a specimen to resist capture stress for longer periods (Braccini & Waltrick, 2019; Grant & Campbell, 2020; Morón‐Elorza et al., 2022). The effect of body size may also explain the lower probability of AVM detected in deeper fishing operations for *G. melastomus*, where larger specimens were sampled.

The stabilization in body size suggested an indirect relationship to maturity because AVM probability stabilized at sizes that corresponded to the approximate lengths at which these species reach maturity (Ramírez‐Amaro et al., 2020). Immature sharks generally exhibit higher standard metabolic rates compared with mature specimens, as previously reported for *S. canicula* among other species (Molina et al., 2020; Sims, 1996). Consequently, immature sharks may consume more oxygen during fight responses, requiring increased breathing rates and glucose volumes to cope with stress, making them more susceptible to AVM. Considering that AVM probabilities have been reported to be higher for pregnant females and males during the reproductive period in other chondrichthyan species (Prado et al., 2022; Wosnick et al., 2019), future research should explore the inclusion of noninvasive technologies to provide further insight into the particular reproductive status of mature specimens.

### Environmental conditions

Sudden increases in AVM probability occurred when atmospheric temperature reached 20°C for *S. canicula* and 16°C for *G. melastomus*, representing a temperature change of 6°C and 2.5°C, respectively, from atmospheric conditions to sea bottom temperatures. These findings highlight the greater vulnerability of the deepwater species to thermal stress. As ectothermic species, exposure to elevated temperatures leads to important changes in their core temperature (Prohaska et al., 2021). Such changes can induce metabolic acidosis, threatening the survival of the captured sharks, as observed in the Gummy shark, *Mustelus antarcticus* (Guida et al., 2016). Elevated temperatures have also been related to decreased erythrocyte counts in *S. canicula*, which compromises oxygen transportation (Pegado et al., 2020). Furthermore, temperature increases and pressure changes related to depth may also affect liver oil density and composition, potentially influencing AVM if liver damage is significant, as reported for the Portuguese dogfish (*Centroscymnus coelolepis*) (Priede et al., 2020). It is noteworthy that increasing temperatures associated with climate change scenarios may exacerbate AVM risks for these ectothermic species (Wagner et al., 2023).

Our results also indicated that the interplay between body size and temperature is crucial in predicting the probability of AVM for *S. canicula*. Larger specimens exhibited greater resilience to thermal stress. This may be a consequence of an increased insulation capability provided by their larger size (Prohaska et al., 2021; Stevens & Sutterlin, 1976), as well as of having increased energy stores with which to face the stress (Braccini & Waltrick, 2019; Skomal & Mandelman, 2012).

Cloud coverage and particularly wind force also had a weak influence in the models. Increased cloud coverage led to a decrease in AVM for *S. canicula*, likely because greater cloud cover reduces thermal stress and desiccation by protecting the sharks from direct solar radiation while on deck (Revill et al., 2005). Increased wind strength can ease the desiccation of the specimens and augment the magnitude of the vessel shaking, causing the sharks to collide and injure itself (Revill et al., 2005). However, these environmental parameters are difficult to standardize because they depend on the vessel settings and handling practices, such as the presence of structures providing shade, time of day, and the vessel direction relative to wind or waves.

### Fishing operations

The probability of AVM increased with time on deck for both species and became pronounced after 15 min. Such an increase was gradual for *S. canicula* and more rapid for *G. melastomus*. A significant number of the latter were found dead when gear was retrieved or shortly after exposure on deck. In contrast, *S. canicula* showed greater resilience, particularly among larger specimens surviving for extended periods. Once on deck and unable to breathe, sharks experience respiratory acidosis, which may be compounded by metabolic acidosis from physical activity and increased temperature. These factors together can often lead to AVM (Shuttleworth, 1985; Skomal & Mandelman, 2012). Increased gravity force while on deck can also damage internal organs, which are only held by connective tissues and not protected by a rigid skeleton (Poisson et al., 2014). Handling processes, such as lifting the specimens by head or caudal fin or piling them together can further harm organs and tissues (Poisson et al., 2014). A large interaction between time on deck and temperature was observed. The probability of AVM remained high at elevated temperatures, even with brief periods of exposure on deck. In contrast, large specimens had lower AVM probabilities than small specimens, even at the longest periods on deck, highlighting their enhanced resilience to stress.

The total biomass in the cod‐end of the trawl had a minor but notable effect on the AVM probability for both species, with higher biomass increasing AVM risk. The crushing force resulting from a large biomass can damage shark organs and hinder respiration during the tow (Revill et al., 2005). However, the extent of the trauma may vary based on the position of the particular shark in the cod‐end, timing of capture, and composition of the catch (e.g., presence of spines, teeth, rocks) (Rodríguez‐Cabello et al., 2005). This variation hampered the standardization of this parameter to predict mortality.

### Implications for management

Bycatch mitigation efforts in EU fisheries often overlook those biological, environmental, and fishing‐related factors that make certain specimens or species more vulnerable to AVM (Gilman et al., 2024; Giovos et al., 2024; Koehler et al., 2025). We identified common AVM drivers for *S. canicula* and *G. melastomus* across these factors, with the latter exhibiting higher vulnerability. Given that phylogenetic relatedness and shared physiological or ecological traits influence AVM risk—causing different species to exhibit similar responses—our insights may provide valuable information for precautionary measures for more threatened species (Dapp et al., 2016; Gilman, Chaloupka, et al., 2022). Accordingly, we provide a series of recommendations tailored to the AVM risks identified for *S. canicula* and *G. melastomus* and offer guidance for related species, particularly in data‐limited contexts. These recommendations may serve as a foundation for bycatch mitigation strategies in the context of the current EU Action Plan for sustainable fisheries and have potential for broader fisheries management efforts (European Commission, 2023; Gilman et al., 2024; Koehler et al., 2025).

Bycatch avoidance is the most effective strategy for reducing fisheries‐induced mortality (Ellis et al., 2024). We found that AVM risk was higher for smaller size classes, particularly under elevated atmospheric temperatures. Integrating biological and environmental factors into marine spatial planning may enhance its effectiveness in reducing bycatch mortality. This may involve adjusting fishing practices to cooler periods, such as nighttime or early morning, especially during warmer seasons, and targeting closures on nursery areas with high abundances of smaller individuals to mitigate AVM risk. When avoidance is not feasible, bycatch reduction devices to enable escape, particularly the use of excluder grids at trawl entrances, offer an alternative with demonstrated potential for bycatch reduction (Ellis et al., 2024; Huang et al., 2024). Targeted use of excluder grids in specific areas and periods of heightened AVM risk, as those identified above, could further reduce capture rates and mitigate mortality (Brčić et al., 2015; Campbell et al., 2020).

When avoidance and escape strategies are impractical, minimizing postcapture mortality becomes crucial. Our findings emphasize that immediate release notably reduces AVM. Ramps and other release systems may be particularly beneficial for handling large specimens and minimizing release times (Murua et al., 2022). Handling and release measures should prioritize the species and size classes most vulnerable to AVM for targeted implementation. Given the nonbinding nature of most of the current handling and release guidelines, national fisheries authorities, RMFOs, and international agencies, including the EU, can play a key role in developing mandatory policies targeting the quick and safe release of vulnerable chondrichthyans. Implementation and compliance would then rely on national agencies (Gilman et al., 2024; Giovos et al., 2024; Koehler et al., 2025). Enforcement challenges may be addressed through increased onboard observer efforts and the implementation of remote electronic monitoring (REM) systems (van Helmond et al., 2020). A recent mandate under the EU Common Fisheries Policy aims to implement REM systems across its fleets (European Union, 2023). Additionally, reducing thermal stress by keeping catches cool and avoiding exposure to solar radiation while on deck could enhance survival; installing deck covers or shade structures may offer a practical and low‐cost solution (Talwar et al., 2017).

Although our recommendations, based on *S. canicula* and *G. melastomus*, enhance understanding of AVM drivers and support bycatch mitigation for more threatened species, caution is needed when extrapolating these findings, underscoring the need for further research to validate their broader applicability (Gilman, Chaloupka, et al., 2022). Despite these limitations, the insights gained underscore the importance of considering the spatiotemporal variability of environmental and fisheries operations. Consequently, conservation outcomes may benefit from integrating these recommendations into dynamic ocean management (DOM) frameworks, where management measures adapt across space and time in response to environmental variability and ocean uses (Pons et al., 2022).

The DOM frameworks have been proposed as a more effective alternative to static approaches and to balance bycatch reduction with economic opportunities for fisheries (Ortuño‐Crespo et al., 2024; Pons et al., 2022). Predictive AVM models, like those we developed, combined with tools for estimating capture rates across size classes could forecast high‐risk times and locations. Despite their potential, implementing DOM frameworks requires clear objectives, robust data, and overcoming logistical and economic challenges. Currently, the EU lacks DOM‐based bycatch mitigation strategies, underscoring the need for further research and policy development to assess their feasibility in reducing fisheries‐related mortality (Pons et al., 2022; Vigo et al., 2024).

Chondrichthyan conservation is at a pivotal moment and requires effective strategies to reduce bycatch mortality in threatened species. To address knowledge gaps in bycatch mitigation, we devised a novel machine learning modeling approach to identify key AVM drivers that accounts for interactions and nonlinear responses. We found that temperature, on‐deck time, and body size were the main drivers of AVM for *S. canicula* and *G. melastomus* in a commercial trawl fishery from the western Mediterranean. Critical thresholds associated with substantial increases in AVM were identified, including temperatures above 20°C and 16°C, on‐deck exposure exceeding 15 min, and body sizes below 40 and 55 cm for *S. canicula* and *G. melastomus*, respectively. Moving forward, these findings can support management authorities (e.g., international binding instruments, EU directives, RFMOs, and national agencies) in adopting hierarchical decision‐making approaches for bycatch mitigation. This includes tailoring bycatch avoidance, gear escape, and postcapture mortality reduction measures to specimens, areas, and periods of heightened mortality risk to maximize effectiveness. Insights from these model species may serve to inform precautionary measures for related threatened species. Finally, our modeling framework has global applicability and is a scalable tool to estimate AVM across species and regions under dynamic environmental and fishing conditions and thus offers a valuable resource for resource managers to ensure future sustainability.

## Supporting information



Supplementary Materials.
