# Supplementary material for: Key drivers of at‐vessel mortality in demersal sharks
Source: Conserv Biol. 2025 Jul 3;40(1):e70100. doi: 10.1111/cobi.70100 (PMC12856789; doi:10.1111/cobi.70100)
Supplement: Supplementary file 1 — Supplementary Materials. [file COBI-40-e70100-s001.pdf]

Machine learning reveals key drivers of at-vessel mortality in demersal sharks

David Ruiz-García[1,2], Claudio Barría[2,3], Juan A. Raga[1], David March[1,4]

[1]Unidad de Zoología Marina, Instituto Cavanilles de Biodiversidad y Biología Evolutiva, Universitat de València, Paterna, Spain.

[2]Association for the study and conservation of elasmobranchs and its ecosystems (Catsharks), Barcelona, Spain.

[3]Department of Functional Biology, Genetics, University of Oviedo, Oviedo, Spain.

[4]Centre for Ecology and Conservation, College of Life and Environmental Sciences, University of Exeter, Penryn, UK.

Contents:

Supplementary figures (Supplementary Figure 1-2).
Supplementary tables (Supplementary Table 1-2).

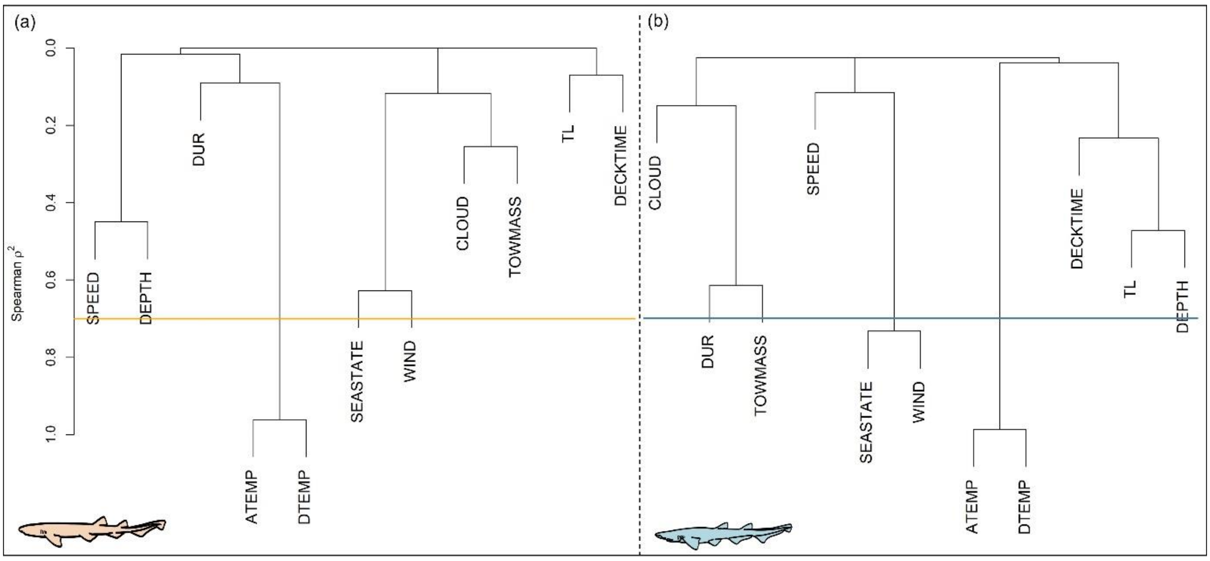

Supplementary Figure S1. Hierarchical cluster on exploratory variables based on squared Spearman correlation. The line represents the 0.7 threshold used for assessing collinearity: (a) *Scyliorhinus canicula*; (b) *Galeus melastomus*. Predictor acronyms and units are described in Table 1.

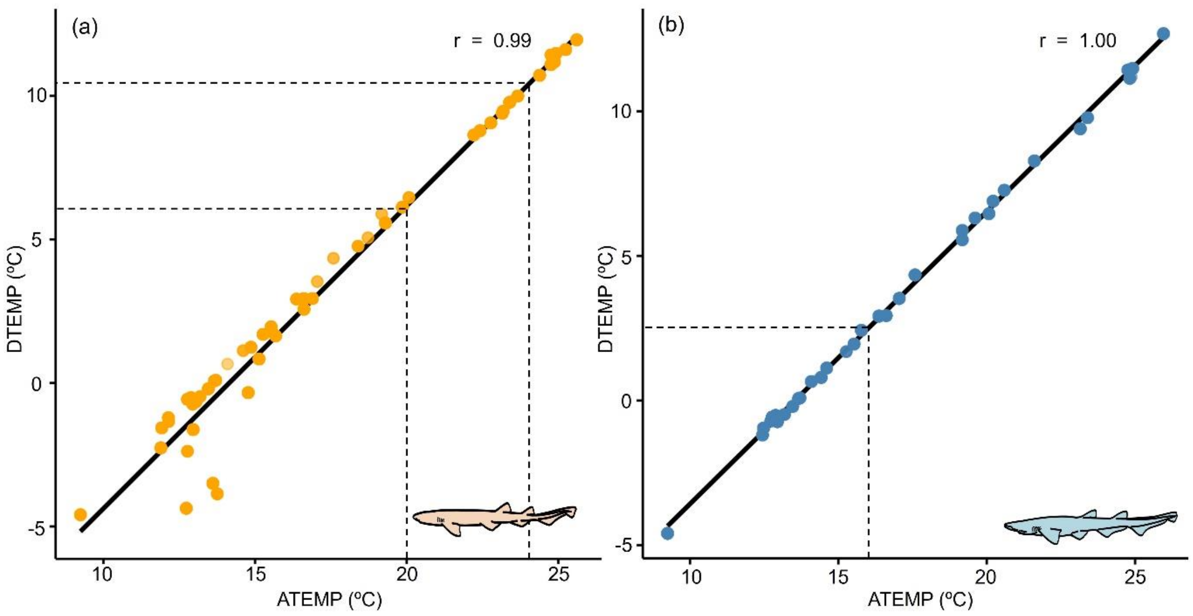

Supplementary Figure 2. Linear relationship between the atmospheric temperature (ATEMP) and the difference between atmospheric and sea bottom temperature (DTEMP) at which each specimen of (a) *Scyliorhinus canicula* and (b) *Galeus melastomus* was exposed in the studied tows. Dashed lines reference the thresholds over which at-vessel mortality rates changed importantly as reflected in the models (Figures 3 and 4).

Supplementary Table S1. Fitted combinations of the boosted regression tree (BRT) parameters used in model optimization for *Scyliorhinus canicula*. Results are ordered from smallest to largest cross-validated deviance. Selected parameters for analysis are marked with a grey shading.

Learning rate	Tree complexity	Bag fraction	Number of trees	Cross-validation AUC	Cross-validation deviance
0.01	5	0.7	550	0.865	0.821
0.01	5	0.6	400	0.866	0.822
0.005	5	0.7	850	0.865	0.824
0.005	5	0.5	800	0.865	0.825
0.001	5	0.6	4250	0.865	0.825
0.01	3	0.7	500	0.860	0.826
0.001	3	0.7	5350	0.861	0.826
0.005	3	0.7	1200	0.861	0.827
0.001	5	0.7	4000	0.864	0.827
0.005	5	0.6	650	0.864	0.827
0.001	3	0.5	5500	0.862	0.830
0.001	3	0.6	5050	0.861	0.830
0.005	3	0.5	1000	0.861	0.830
0.01	3	0.5	500	0.861	0.830
0.001	5	0.5	3800	0.864	0.831
0.005	3	0.6	1000	0.861	0.831
0.01	3	0.6	550	0.859	0.832
0.01	5	0.5	350	0.861	0.836
0.01	1	0.5	1100	0.852	0.840
0.01	1	0.7	1300	0.852	0.841
0.005	1	0.5	2200	0.853	0.841
0.005	1	0.7	2400	0.852	0.841
0.01	1	0.6	1250	0.852	0.842
0.005	1	0.6	2350	0.852	0.844
0.001	1	0.7	10000	0.852	0.845
0.001	1	0.6	9850	0.852	0.845
0.001	1	0.5	9550	0.852	0.846

Supplementary Table S2. Fitted combinations of the boosted regression tree (BRT) parameters used in model optimization for *Galeus melastomus*. Results are ordered from smallest to largest cross-validated deviance. Selected parameters for analysis are marked with a grey shading.

Learning rate	Tree complexity	Bag fraction	Number of trees	Cross-validation AUC	Cross-validation deviance
0.005	1	0.5	1300	0.865	0.769
0.01	1	0.6	850	0.866	0.771
0.01	1	0.5	700	0.867	0.772
0.001	1	0.5	5050	0.865	0.773
0.01	1	0.7	600	0.864	0.778
0.001	1	0.6	4800	0.864	0.779
0.005	1	0.6	1350	0.865	0.779
0.001	1	0.7	5450	0.864	0.781
0.005	1	0.7	1450	0.863	0.782
0.01	3	0.5	200	0.851	0.817
0.001	3	0.5	2600	0.856	0.818
0.005	3	0.6	500	0.854	0.822
0.001	5	0.5	2000	0.854	0.822
0.005	3	0.5	450	0.852	0.822
0.01	3	0.6	250	0.853	0.823
0.01	5	0.5	250	0.855	0.826
0.001	3	0.6	2500	0.850	0.826
0.005	5	0.5	400	0.852	0.827
0.01	3	0.7	300	0.850	0.830
0.005	3	0.7	450	0.851	0.830
0.001	5	0.6	2000	0.850	0.831
0.005	5	0.6	350	0.846	0.831
0.001	3	0.7	2550	0.849	0.833
0.01	5	0.6	200	0.847	0.834
0.01	5	0.7	200	0.848	0.840
0.001	5	0.7	2300	0.847	0.842
0.005	5	0.7	500	0.849	0.844
